# The scientific elucidation of *daodi* medicinal materials

**DOI:** 10.1186/s13020-020-00367-1

**Published:** 2020-08-20

**Authors:** Xindan Liu, Ying Zhang, Menghua Wu, Zhiguo Ma, Zihan Huang, Fang Tian, Sihan Dong, Simin Luo, Yu Zhou, Jinju Zhang, Nanxin Li, Xiaofang He, Hui Cao

**Affiliations:** 1 Research Center for Traditional Chinese Medicine of Lingnan (Southern China), Jinan University, Guangzhou, 510632 China; 2 College of Pharmacy, Jinan University, Guangzhou, 510632 China

**Keywords:** *Daodi* medicinal materials, Chinese medicinal materials, Ecological environment, Chemical components, Pharmacological functions

## Abstract

*Daodi* medicinal materials (DMMs), with unique characteristics and specific ecological growing environments, are recognized as high-quality medicinal products of Chinese medicinal materials (CMMs). The quality evaluation of CMMs is fundamental for standardization. The concept and application of DMMs have a long history as described in records in ancient books and rooted in practice and experience over generations. DMM is the specific term for pure, superior medicinal herbs with the following characteristics: optimum harvest season (reflecting the appropriate developmental stage of the plant), scrupulous processing, traditional preparation technology, etc. As DMM and high-quality medicinal products are traditionally thought to be closely related, modern scientific studies that confirm the association of these products are described. This article aims to clarify the scientific elucidation of DMMs.

## Background

In recent years, as the use of Chinese medicinal materials (CMMs) has increased, the international attention paid to the safety, stability and efficacy of CMMs has increased. Some authentic and superior CMMs that are grown in specific regions and widely recognized as having better therapeutic effects are called *daodi* medicinal materials (DMMs) [1]. DMMs, based on the theory, origin, processing, and prominent curative effect of CMMs, are the essence of Chinese cultural heritage [2]. DMMs were first recorded in *Zhen Zhu Nang Yao Xing Fu* (Precious Drus in Rhyme), a book written 700 years ago. The term “*daodi* medicinal material” is widely found in *Ben Cao Pin Hui Jing Yao* (Essentials of Materia Medica Distinctions), a book compiled by the Imperial Hospital during the Ming Dynasty (1368–1644 A.D.), in which 268 medicinal herbals are listed. The entry “original source” was formally listed under each medicinal herb heading, specifying *daodi* production regions. It has been suggested that the quality of CMMs is highly correlated with their geographical origins. In ancient times, the identification of DMMs was commonly carried out based on the characteristics of the superficies, and this approach depended to a certain extent on empirical experiences and assumptions. Currently, modern scientific analytical techniques may be applied to confirm the validity of associations between high-quality medicinal products and DMM to ensure their utility, clarifying the scientific understanding of *daodi* medicinal materials.

The word “*dao*” (in “*daodi*”) is an ancient Chinese unit of measurement used to divide administrative districts, and this term can be retraced to the Eastern Han Dynasty (25–220 A.D.) as described in *Hou Han Shu* (Book of Later Han, 432–445 A.D.) [3]. In the Tang Dynasty (618–907 A.D.), the nation was divided into 10 “*dao*” according to landscapes in the *Zhenguan* Period, and then the number was increased to 15 “*dao*” in the *Kaiyuan* Period. Currently, “*dao*” is conceptually similar to the modern organizational system of provinces. The word “*di*” (in “*daodi*”) refers to regions and geography. Nowadays, “*daodi* medicinal materials” refers to the distinctively higher quality of the medicinal materials that grow in a certain area.

The establishment of DMMs is related to resources, agricultural technology and CMM development. In ancient China, due to agriculture production, rich experience along with advanced technology in growing and processing medicinal herbals was accumulated, which resulted in the exchange of resources around the world [4]. In addition, the vast territory of China, including plains, hills, mountains, lakes, rivers and seas with different climates, sunshine, soils and ecological environments, provides favorable conditions for the growth of medicinal herbs. Through meticulous selection over the course of production and continuous clinical tests, DMMs have been proven to be definitely curative in medical treatments and therefore have been handed down from generation to generation [5].

DMM can be very effective if proper consideration is given to the characteristics of the original sources, growth and seasonal changes of the material; however, the same medicinal herb grown in different areas does not have the same effectiveness even if they are the same plant. In China, traditional Chinese medicine doctors usually select a superior populations or variety to prescribe based on geographical features. In addition, different harvest times or processing methods can greatly influence the quality of CMMs in terms of chemical components and pharmacological functions. Thus, DMM, as an authentic and superior medicinal product, needs to be validated by modern analytical methods for each CMM. In this paper, the scientific elucidation of DMMs will be introduced, as well as a putative production chain for processing traditional, experience-based CMMs.

## *Daodi* medicinal materials are the essence of Chinese cultural heritage

Over the long course of clinical selection, by discarding the inferior and retaining the superior, DMMs have been acquired. DMMs represent the influence of populations, together with optimum harvest season, habitat and growing conditions such as sunshine, soil, and water. Generally, the identification of DMMs is a useful tool for the quality evaluation of CMMs.

### Populations

**Fig. 1:**
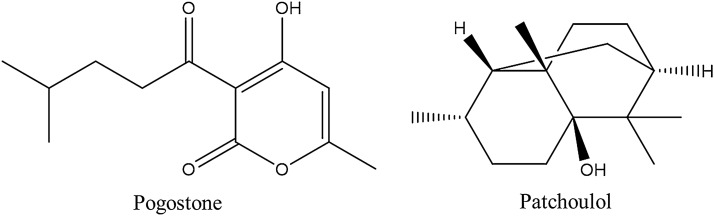
Structures of the pogostone and patchoulol in *Pogostemon cablin*

**Fig. 2:**
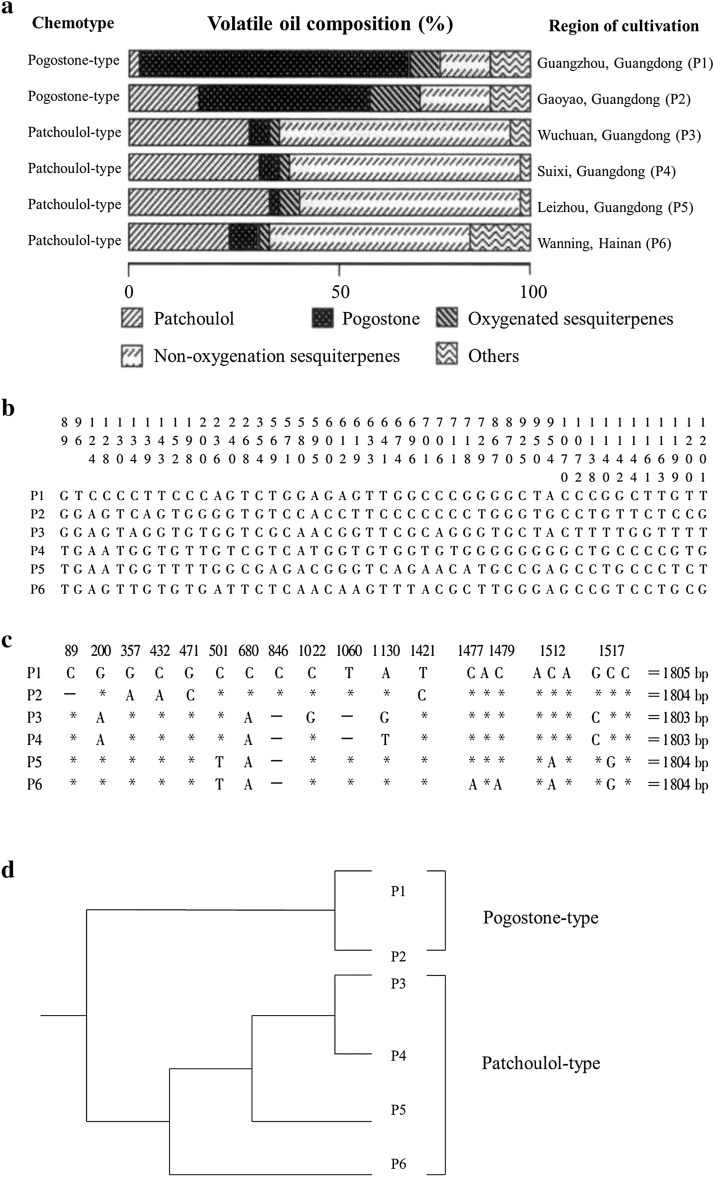
Volatile oil composition in the leaves (**a**), comparison of the variable sites in the *mat*K sequence (**b**), comparison of the variable sites in the 18S rRNA sequence (**c**) and cluster trees of both the *mat*K and 18S rRNA gene sequences (**d**) of *Pogostemon cablin* (Blanco) Benth. from 6 different locations. The top number indicates the nucleotide position upstream of the *mat*K (**b**) and the 18S rRNA (**c**) sequence, an asterisk (*) indicates the same nucleotide as the P1 sequence, and a hyphen (—) indicates an alignment gap (**c**)

**Fig. 3:**
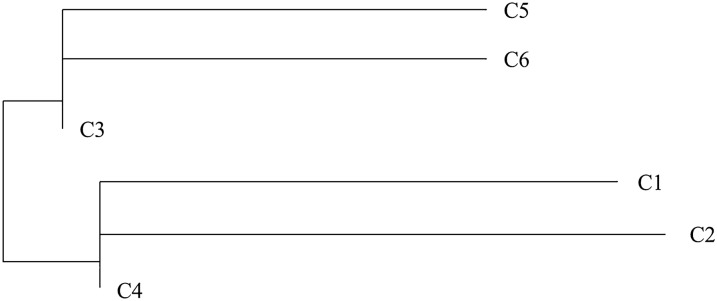
Phylogenetic tree of the *mat*K gene sequence (E) of *Cnidium monnieri* (L.) Cuss. from six locations (C1–C6)

Different growing environments may greatly influence the quality and chemical components of closely related populations. Modern experimental research, especially molecular biological identification, has validated that the different production areas of CMMs are closely related to the quality and DNA sequence divergence of CMMs [6–8]. For example, as the dominant constituents, patchoulol and pogostone are the basis for the anti-inflammatory activity of *Pogostemon cablin* (Blanco) Benth. [9]. And it has been found that pogostone also exhibits potent anti-fungal [9], antiapoptotic [10], antioxidant [11], and immunosuppressive [12] properties. In our previous study, *P. cablin* produced in Shipai (in Guangzhou city, Guangdong province, SP) and Gaoyao (in Zhaoqing city, Guangdong province, GY) differed from *P. cablin* cultivated in Hainan province (HN) and Zhanjiang city (in Guangdong province, ZJ) not only in the total amount of volatile oil but also in genotype [13–15]. According to the composition of the volatile oil, *P. cablin* is divided into two chemotypes: SP and GY cultivars belong to the pogostone-type, while the HN and ZJ cultivars belong to the patchoulol-type (Fig. 1). Moreover, we have demonstrated that the sequence divergence of both the *mat*K and 18S rRNA genes among 6 samples of *P. cablin* from different locations was well correlated with the regions of cultivation and intraspecific essential oil chemotypes (Fig. 2) [16]. The same is true for other medicinal herbs. The major pharmacological components in *Cnidium monnieri* (L.) Cuss. are coumarins [17]. Similarly, according to the coumarin chemotypes, *C. monnieri* has been classified into three chemotypes, the osthol-linear furanocoumarins-type (chemotype I), principally cultivated in regions of Jiangsu and Hunan provinces; the angular furanocoumarins-type (chemotype II), mainly produced in Heilongjiang province; and the transition-type (chemotype III), largely came from Henan and Hebei regions [18]. It was found that there were 12 variable sites in the *mat*K gene sequence of *C. monnieri* from different populations. A phylogenetic tree constructed by the neighbor-joining (NJ) method showed that the phylogenetic relationship of 6 *C. monnieri* cultivars was well correlated with their geographical distribution and intraspecific coumarin chemotypes of (Fig. 3) [19]. Likewise, Laboratory research showed a clear correlation between the rDNA ITS sequence and the phenotype of *Dendrobium officinale* Kimura et Migo from different populations [20]. The phylogenetic relationship predicted by the 5S rRNA spacer region data correlated well with the essential oil chemotype of *Acorus calamus* L. collected from various locations [21]. However, the difference in the alkaloid content of *Fritillaria thunbergii* Miq. from various habitats did not result from variation in the 5S rRNA sequence but from the microenvironment [22]. It’s reported that 5S rRNA is highly-conserved arcoss all species [23], thus, different environments did not produce changes in 5S rRNA of different *F. thunbergii* populations, but produced differences in secondary metabolites. In these cases, it can be concluded that genetic diversity existed among different populations is relevant to the cultivation regions except some highly-conserved DNA markers.

### Harvest time

CMMs harvested during different growing periods contain different plant metabolites. For instance, the content of essential oil increased as the age of *P. cablin* increased: patchoulol, *α*-bulnesene and other sesquiterpenes accumulated to high concentrations at 210 days after maturation [24]. According to the report from Jin et al. [25], *Chaenomeles speciosa* (Sweet) Nakai produced the highest quality yields when harvested in early July. In the case of *Desmodium styracifolium* (Osb.) Merr., the best collection season was early October, when the highest concentrations of polysaccharides, flavonoids and schaftoside were measured [26].

### Sunshine

Sunshine is an important factor for the formation of DMMs. For example, the content of volatile components in *Houttuynia cordata* Thunb. was closely related to light intensity: monoterpenoids and nonterpenoids were positively and negatively associated with light intensity, respectively [27]. In *Viola yedoensis* Makino, the content of flavonoids and coumarins was positively correlated with light intensity [28]. Although it has been demonstrated that higher concentrations of total patchoulol corresponded to lower light intensity in *P. cablin*, there was no statistically significant correlation between patchoulol content and shade [29]. In recent years, circadian clocks that temporally organize many aspects of growth and metabolism have even been found in numerous plant species [30–32]. For example, in *Antirrhinum majus* L., *monoterpene synthase* mRNA levels and corresponding monoterpene emission, which followed diurnal rhythms, were controlled by a circadian clock [33]. A similar daily fluctuation was found in the endogenous level of geranyl acetate and in the expression of its biosynthetic gene, *alcohol acetyl transferase* in *Rosa rugosa* Thunb. [31].

### Soil

Soil is also important for the identification of DMMs and for the evaluation of quality. Modern pharmacologic studies have proven that the specific composition of soil has a large influence on the quality and quantity of the chemicals in medicinal herbs. For example, soil available iron (Fe) could promote the accumulation of flavonoids, while soil available manganese (Mn), total potassium (K), and available K had an inhibitory effect on flavonoid content in *Spatholobus suberectus* Dunn [34]. In *Citrus grandis* ‘Tomentosa’, the content of soil available copper (Cu), zinc (Zn), Mn, boron (B), and molybdenum (Mo) was positively associated with flavonoid concentrations and naringin concentrations [35]. There was a positive correlation between soil total nitrogen (N), available K and emodin in *Polygonum cuspidatum* Sieb. et Zucc. [36]. Soil Mn was a favorable factor for accumulating schisantherin A in *Schisandra sphenanthera* Rehd. et Wils., as a significant correlation was also found between these factors [37].

### Water

Another matter regarding DMMs that warrants attention is water. As shown in *Aconitum carmichaelii* Debx., the heavy metals cadmium (Cd), arsenic (As), mercury (Hg), and lead (Pb) concentrations in it were positively associated with the water in Fujiang River (*p *< 0.05) [38]. This result indicates that the quality of CMMs can also be affected by water. To meet the growing demand of DMMs, additional detailed studies should be undertaken in this field.

### Comprehensive ecological factors

Comprehensive research on the relationship between different ecological environment factors and the quality of CMMs has also been recorded. For example, in *Scutellaria baicalensis* Georgi, most of the chemical constituents were negatively correlated with latitude and positively correlated with temperature. Generally, the contents of 21 chemical constituents were higher at low latitudes than at high latitudes. By gradual regression analysis, it was found that the content of baicalin in *S. baicalensis* was negatively correlated with latitude. Similarly, the content of inorganic elements in soil was excessively high (magnesium (Mg) and calcium (Ca) excluded), which had a negative effect on the accumulation of chemical constituents in *S. baicalensis* [39, 40]. Taking the well-known antioxidant herb *Panax ginseng* C. A. Mey. as another example, low temperature was a favorable factor for the accumulation of ginsenosides, as a negative correlation was found between temperature and ginsenoside contents within a certain temperature range, while the levels of soil available B, effective Fe and available N were positively correlated to ginsenoside contents [41]. In recent years, due to overexploitation, the destruction of the ecological environment and the lack of proper cultivation practices, the geographical distributions of most DMMs may undergo large changes. For example, although the Changzhi region of Shanxi province and provinces of north-east China was *P. ginseng*’s original production center, the present production center of it is in Xiaoxinganling region (in Heilongjiang province) and Changbaishan region (in Jilin province) [42]. Similarly, *Panax notoginseng* (Burk.) F. H. Chen historically came from the Tianzhou region (in Guangxi province), but now the dominant medicinal material comes from Wenshan region (in Yunnan province) [43]. In these cases, predicting the geographical distribution of CMMs is important for resource conservation and regional management. Therefore, a geographic information system based on a computer program (TCMGIS) was developed to predict the distribution of CMMs. By integrating geographic location, climate and soil type databases, TCMGIS was able to determine the impacts of environmental components and predict the large-scale distribution of target medicinal herbs such as *P. cablin* [44], *Artemisia annua* L. [45], *Polygonum multiflorum* Thunb. [46], *Morinda officinalis* How [47], *Aquilaria sinensis* (Lour.) Gilg [48], *Rheum tanguticum* Maxim. ex Balf. [49], *Amomum villosum* Lour. [50], etc.

## Traditional descriptions of *daodi* medicinal materials by famous physicians in ancient China

In the use of CMMs, a large emphasis has been placed on the identification of DMMs since ancient times. As recorded in *Shen Nong Ben Cao Jing* (The Divine Shennong’s Classic of Materia Medica, 25–220 A.D.) [51], “each medicinal material has laws for its production region, authenticity, and freshness.” In various chapters of that book, locations in ancient kingdoms and regions, such as mountain valley, river valley or marshes, were mentioned for the first time as medicinal herbs sources. This record indicates that different CMMs come from certain specific areas. In his immense book *Ben Cao Jing Ji Zhu* (Collection of Commentaries on the Classic of the Materia Medica, 480–498 A.D.) [52], Tao Hongjing, a well-known physician in the Northern and Southern Dynasties (420–589 A.D.), used such terms “good”, “quite good”, “fairly good”, “excellent” and “best” to describe the effects of over 40 medicinal herbals commonly used in medical treatment. Moreover, correlations between the sources, developmental stages and efficacy of these medicinal herbs were described. Sun Simiao, a famous physician and pharmacologist of the Tang Dynasty (618–907 A.D.), stated the following in his book *Qian Jin Yi Fang* (Formulas Worth a Thousand Gold Pieces, 682 A.D.) [53]: “Medicinal herbs used by ancient physicians were always from designated original sources, which accounted for their great effectiveness in medical treatment”. In that book, he comprehensively sorted 519 DMMs and systematically stipulated 133 regions of production. According to Kou Zongshi, a famous physician of the Song Dynasty (960–1279 A.D.), in his book *Ben Cao Yan Yi* (Extension of the Materia Medica, 1116 A.D.), “in prescribing medicinal herbs, care should always be taken to select those from proper sources to ensure their effectiveness”, greatly emphasizing the designated original sources of medicinal herbs [54]. During the Jin and Yuan Dynasties (1115–1368 A.D.), the text *Yong Yao Fa Xiang* (Medication Method, 1249 A.D.) also suggested that one could achieve excellent treatment results only by using DMMs with proper production regions and harvest time. Then, the Ming Dynasty (1368-1644 A.D.) document *Ben Cao Meng Quan* (Materia Medica Companion, 1565 A.D.) stated that “the effect will be definitely different if medicinal materials are produced in a different environment” [55]. The record in *Yi Xue Yuan Liu Shi* (Origins of Medicine, 1767 A.D.) attached similar importance to the use of DMMs collected from certain original sources [56]. All of this historical literature showed that the use of DMMs has been a practice since ancient times.

## The scientific elucidation of *daodi* medicinal materials

**Fig. 4:**
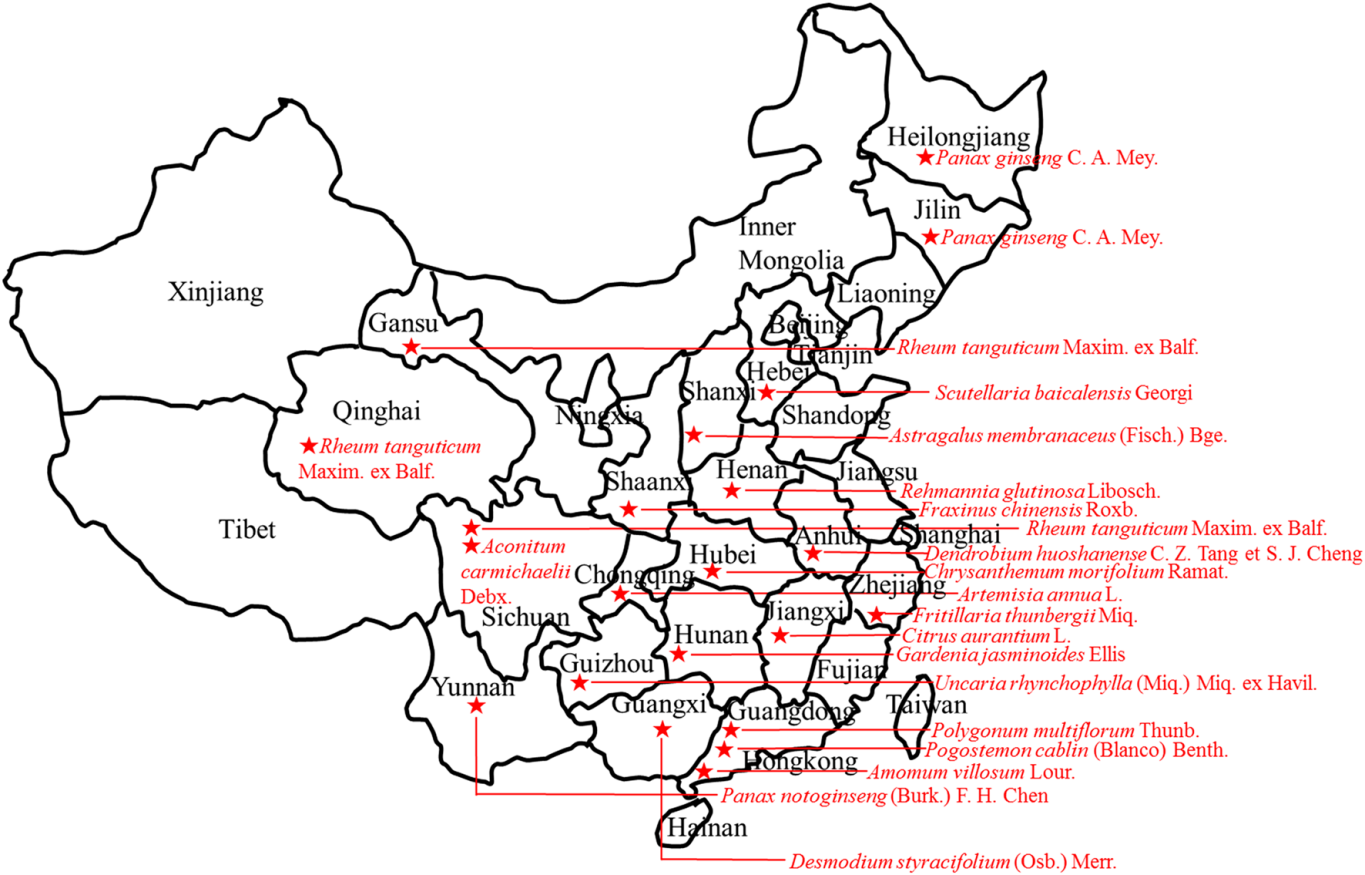
The distribution of some *daodi* medicinal materials in China

DMMs are the subset of CMMs that meet the highest quality criteria. DMMs are not only associated with specific geographic regions (Fig. 4) but also linked to the chemical components and pharmacological function of CMMs.

### Chemical components

**Fig. 5:**
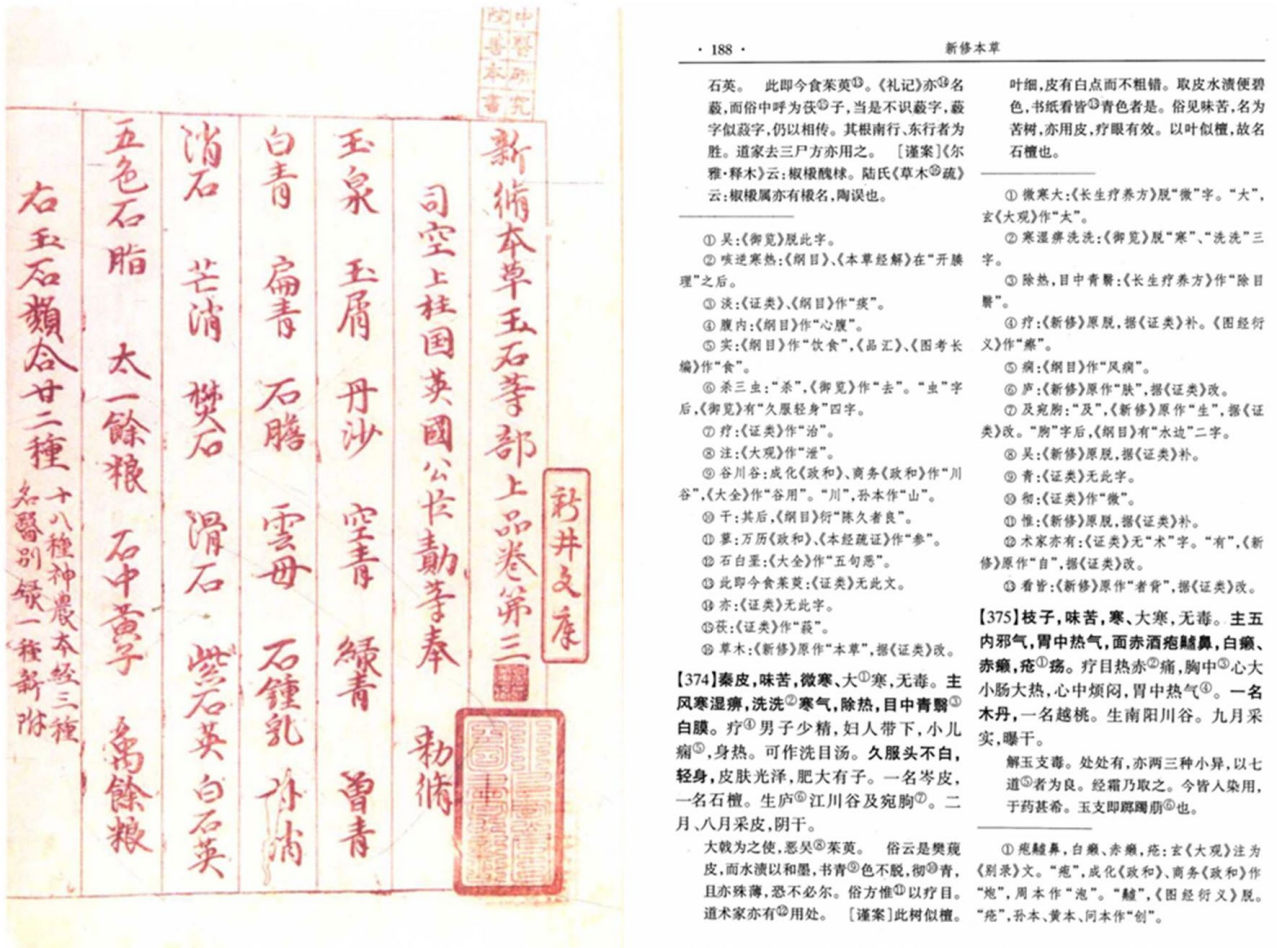
The herbal classic *Xin Xiu Ben Cao* (Newly Revised Materia Medica) describes the metachromatism-based quality evaluation of “*qin pi*” (*Fraxinus chinensis* Roxb.)

As we described above, ecological environments such as topography, sunshine, soil, and water directly influence the secondary metabolites (many of which are bioactive components) in medicinal herbs. The many names of DMMs reflect the connotations of production regions; for instance, “*qin pi*” (*Fraxinus chinensis* Roxb.), “*fen qi*” (*Astragalus membranaceus* (Fisch.) Bge.), “*huai di huang*” (*Rehmannia glutinosa* Libosch.) and “*ba dou*” (*Croton tiglium* L.), where “*qin*”, “*fen*”, “*huai*”, and “*ba*” refer to the names of regions used over the course of ancient Western Zhou Dynasty (1046–771 B.C.) [1]. Modern experimental research has validated that DMMs growing in a certain production region are often of high quality (Table 1). For example, the ancient Chinese medicine book *Xin Xiu Ben Cao* (Newly Revised Materia Medica, 659 A.D.) [57] said that “*Fraxinus chinensis* Roxb., which can change the color of water to a fluorescent color after soaking, is thought to be superior in quality” (Fig. 5). Currently, scientific evidence supporting the rational for such description is available. *F. chinensis* produced from Shaanxi province has a higher content of aesculin and aesculetin than that produced in Sichuan province and Liaoning province, and its stronger fluorescence reaction is consistent with the description written in ancient times [58, 59]. The same observation is true for *Astragalus membranaceus* (Fisch.) Bge. This herb is principally cultivated in a region in Shanxi province, and the cultivar produced in this region contains more astragaloside than do cultivars produced in Shandong, Inner Mongolia, Hebei and Jilin provinces [60]. It is generally recognized that *Rehmannia glutinosa* Libosch. cultivated in Henan province is of particularly high quality. Modern experimental studies have demonstrated that higher levels of the active constituent catalpol content are present in *R. glutinosa* grown in this region than in cultivars grown in areas of Xianyang (in Shaanxi province) and Dali (in Shaanxi province) [61]. Similarly, *A. villosum* cultivated in Yangchun (in Guangdong province) is believed to be superior in quality. The effective medicinal elements in *A. villosum* is bornyl acetate. Modern experimental research has validated that Yangchun-cultivated *A. villosum* has the highest effective component content among different populations [62–64]. In other example, *P. cablin* cultivated in Shipai (in Guangzhou city, Guangdong province) is of particularly high quality. It produced higher levels of the active constituent pogostone than that cultivated in Gaoyao (in Zhaoqing city, Guangdong province), Leizhou (in Zhanjiang city, Guangdong province), Wuchuan (in Zhanjiang city, Guangdong province) and Hainan province [65–70]. Interestingly, the same is true for toxicology. *A. carmichaelii* produced from plantation sites at Jiangyou county of Sichuan province is believed to be superior in quality. The proportions of the major bioactive constituents monoester alkaloids to toxic constituents diester alkaloids amount among 5 samples of *A. carmichaelii* from different localities were well correlative with their regions of cultivation. The highest proportion occurred in cultivar Jiangyou (in Sichuan province), followed by cultivars Hanzhong (in Shaaxi province), Butuo (in Sichuan province), Weishan (in Yunnan province), and Anxian (in Sichuan province) [71].

**Table 1:** The contents (%) of active constituents in different Chinese medicinal materials in *daodi* production region and non-*daodi* production regions

No.	CMM	Active constituents	Content (%)					References
			*Daodi* production region	Non-*daodi* production region				
1	*Fraxinus chinensis* Roxb.	Aesculin, aesculetin	Shaanxi: aesculin (4.37%), aesculetin (1.92%)	Sichuan: aesculin (1.10%), aesculetin (0.21%)		Liaoning: aesculin (2.21%), aesculetin (0.19%)		[58, 59]
2	*Astragalus membranaceus* (Fisch.) Bge.	Astragaloside	Shanxi: 0.37%	Shandong: 0.12%	Inner Mongolia: 0.11%	Hebei: 0.29%	Jilin: 0.32%	[60]
3	*Rehmannia glutinosa* Libosch.	Catalpol	Henan: 0.76%	Xianyang, Shaanxi: 0.34%		Dali, Shaanxi: 0.33%		[61]
4	*Amomum villosum* Lour.	Bornyl acetate	Yangchun, Guangdong: 65.82%	Gaozhou, Guangdong: 55.95%	Guangxi: 61.75%	Yunnan: 50.92%	Burma: 60.71%	[62–64]
5	*Pogostemon cablin* (Blanco) Benth.	Pogostone	Shipai, Guangzhou, Guangdong: 68.43%	Gaoyao, Zhaoqing, Guangdong: 26.15%	Leizhou, Zhanjiang, Guangdong: 4.78%	Wuchuan, Zhanjiang, Guangdong: 5.20%	Hainan: 8.97%	[65–70]

*CMM* Chinese medicinal material

### Pharmacological functions

Pharmacological functions are actually the outside manifestations of CMMs. In the case of DMMs, the conditions in a certain region are thought to confer clinical superiority, and for this reason, DMMs are considered the most efficacious among CMMs [72]. Scientific evidence supporting the alleged clinical superiority of DMMs is the subject of ongoing research. For example, *Dendrobium huoshanense* C. Z. Tang et S. J. Cheng produced in Huoshan (in Anhui province) is considered to be superior in quality. Accordingly, the hepatoprotective effect is the best for the Huoshan cultivar, second for the Yunnan cultivar, and last for the other region cultivars [73]. *P. cablin* has been classified into two chemotypes, the patchoulol-type, including cultivars HN and ZJ, and the pogostone-type, including cultivars SP and GY. Accordingly, this cultivars produced in GY are more potent than those from ZJ in terms of promoting digestion [74] and antibacterial [75] effects. In addition, the toxicity of DMMs is often less potent than that of non-DMMs. For instance, *P. multiflorum* is principally cultivated in region Deqing county of Guangdong province, which has the largest output and the longest history of medicinal use. Accordingly, cultivar Deqing showed less potent cytotoxicity than cultivar Chongqing in HepG2 and LO2 cells [76]. These studies show considerable promise for explaining the scientific mechanism of DMM superiority.

## *Daodi* medicinal materials are the basis of the medicinal industry and clinical practice

**Fig. 6:**
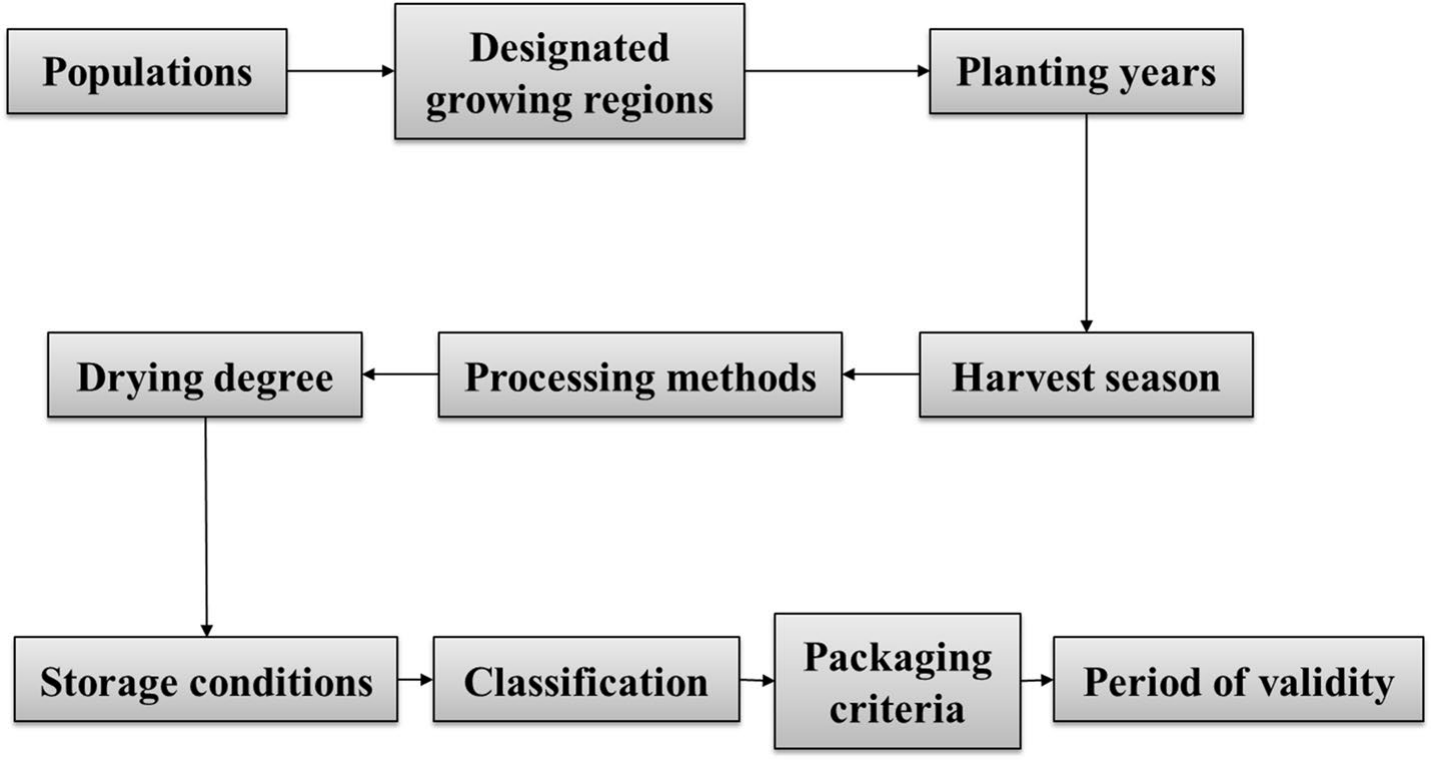
A putative production chain for CMMs

CMMs are the materials processed into decoction ingredients or used to produce proprietary drugs. The identification of DMMs is important in quality evaluation and disease treatment. In addition to the optimum harvest season, the processing and standard prescription of CMMs produce the unique characteristics of DMMs, and a plausible production chain of CMMs is hypothesized in Fig. 6. The production chain of CMMs is based on the content we described above, including populations, designated growing regions (*daodi*), and harvest season, as well as the extensive quality control knowledge accumulated for CMMs by Liu et al. [77–80]. Compared to previous production chain of CMMs [77–80], factors such as populations, designated growing regions and harvest time directly influenced the quality of CMMs were emphasized. Every procedure in the production chain should be standardized to guarantee the prominent curative effect of medicinal materials. For example, the manufacturing procedure for *Pinellia ternata* (Thunb.) Breit. was standardized by orthogonal design [81]. In addition, modern analytical methods have revealed the processing mechanism of many CMMs, including *Coptis chinensis* Franch. [82–84], *Xanthium sibiricum* Patr. [85–87], *Siegesbeckia orientalis* L. [88, 89], *Descurainia sophia* (L.) Webb. ex Prantl. [90, 91], *Cassia obtusifolia* L. [92, 93], etc. Additionally, cooperation among universities, research institutes and pharmaceutical manufacturers should be strengthened to communicate information regarding CMMs.

## Conclusions

DMMs have long maintained, currently have, and will continue to maintain a good reputation on the basis of their excellent curative effects. In our review, DMM is the specific term for pure, superior medicinal herbs with the following characteristics: optimum harvest season (reflecting the appropriate developmental stage of the plant), scrupulous processing, traditional preparation technology, etc. Historical literature, modern phytochemical and pharmacological methods have provided additional scientific data and a theoretical basis to validate the mechanisms of DMMs. In addition, every procedure in the production chain of CMMs should be standardized to guarantee the prominent curative effect of medicinal materials. Effectively establishing a correlation among the active components, clinical efficacy and identity of DMMs is an important aspect in the quality evaluation of CMMs. The core scientific elucidation of DMMs should be continuously carried out, and multidisciplinary measures should be adopted to explore scientific and practical methodologies for the further research of DMMs.

## Data Availability

Not applicable.

## Abbreviations

DMMs: daodi medicinal materials
CMMs: Chinese medicinal materials
SP: Shipai (in Guangzhou City, Guangdong province)
GY: Gaoyao (in Zhaoqing City, Guangdong province)
HN: Hainan province
ZJ: Zhanjiang City (in Guangdong province)
NJ: neighbor-joining
Fe: iron
Mn: manganese
K: potassium
Cu: copper
Zn: zinc
B: boron
Mo: molybdenum
N: nitrogen
Cd: cadmium
As: arsenic
Hg: mercury
Pb: lead
Mg: magnesium
Ca: calcium
TCMGIS: geographic information system based on a computer program
